# Camera–Monitor Systems as An Opportunity to Compensate for Perceptual Errors in Time-to-Contact Estimations

**DOI:** 10.3390/vision7040065

**Published:** 2023-10-08

**Authors:** Elisabeth Maria Wögerbauer, Heiko Hecht, Marlene Wessels

**Affiliations:** Department of Psychology, Johannes Gutenberg-University Mainz, 55122 Mainz, Germany; hecht@uni-mainz.de (H.H.); mwessels@uni-mainz.de (M.W.)

**Keywords:** time-to-collision estimation, acceleration, camera–monitor systems

## Abstract

For the safety of road traffic, it is crucial to accurately estimate the time it will take for a moving object to reach a specific location (time-to-contact estimation, TTC). Observers make more or less accurate TTC estimates of objects of average size that are moving at constant speeds. However, they make perceptual errors when judging objects which accelerate or which are unusually large or small. In the former case, for instance, when asked to extrapolate the motion of an accelerating object, observers tend to assume that the object continues to move with the speed it had before it went out of sight. In the latter case, the TTC of large objects is underestimated, whereas the TTC of small objects is overestimated, as if physical size is confounded with retinal size (the size–arrival effect). In normal viewing, these perceptual errors cannot be helped, but camera–monitor systems offer the unique opportunity to exploit the size–arrival effect to cancel out errors induced by the failure to respond to acceleration. To explore whether such error cancellation can work in principle, we conducted two experiments using a prediction-motion paradigm in which the size of the approaching vehicle was manipulated. The results demonstrate that altering the vehicle’s size had the expected influence on the TTC estimation. This finding has practical implications for the implementation of camera–monitor systems.

## 1. Introduction

Camera–monitor systems (CMS, the replacement of the traditional rear-view mirror) constitute an opportunity to save gas and to cater to drivers’ need to monitor rearward traffic. Unlike traditional rear-view mirrors, CMS have the potential to revolutionize the rearward viewing conditions of drivers. They provide novel degrees of freedom to optimize the driver’s viewing axis and the driver’s field of view (e.g., [1,2]). This has the potential to improve the rearward view of drivers and consequently reduce lane-change crashes, which account for up to 10% of all crashes reported on U.S. roadways [3,4]. CMS also offer the opportunity to enhance the mirror image in ways that extend far beyond the optimization of the driver’s view port. In principle, one can enhance the camera-based view of the rearward scene by modifying the objects within this displayed scene. Whereas convex or bi-focal mirrors can only magnify, minify, or distort a given camera image, the CMS is amenable to image manipulation. For instance, with an object-recognition software, cars approaching from the rear can be readily recognized, as is evident in the current, state-of-the-art efforts in autonomous driving. Once recognized, the approaching car could be modified in the monitor, which the driver consults in lieu of a traditional rear-view mirror. In the current study, we investigated one particular modification that could be a candidate for improving the accuracy of time-to-contact (TTC) estimations and could thus promote safer driving behavior.

TTC is defined as the time it will take for a moving object to reach a specific location [5,6]. Observers typically perform well when catching a ball or when making a street-crossing decision on the basis of optical TTC information. However, this ability may be challenged when viewing objects through a mirror [7]. The temporal decision as to when exactly to close the hand or to start running can be based on optical information that does not require the direct assessment of the approaching object’s velocity. More precisely, according to tau theory (see e.g., [8]), the relative optical expansion of the object can be exploited to make accurate TTC judgments, provided that the object approaches the observer at a constant velocity. However, as soon as the approaching object no longer moves at a constant speed but accelerates or decelerates, observers fail to adequately take the positive or negative acceleration into account, as would be necessary for an accurate TTC estimation or interception [9,10,11,12,13,14,15,16,17,18,19,20]. In other words, when an accelerating object is occluded, rather than adequately incorporating acceleration as second-order information, the TTC is estimated based on first-order (i.e., velocity) information only. Using a first-order estimation strategy, observers mistakenly assume that the object continues to move at a constant velocity [21]. As a result, the impending TTC is typically overestimated for positively accelerating objects and underestimated for negatively accelerating (decelerating) objects. Such misestimations can have severe consequences for traffic. Suppose driver A plans to overtake a vehicle on the highway but sees an approaching vehicle in the passing lane in the rearview mirror. Driver B in the passing lane recognizes the overtaking intention of Driver A and either slows down to yield or accelerates to quickly move out of the way. Driver A could recognize the yielding intention and overtake [22]. However, if this is not the case and Driver A does not adequately consider the negative acceleration in her TTC estimation, then Driver A is likely to hesitate to overtake because she estimates the TTC of the approaching vehicle to be shorter than it actually is. This TTC underestimation could have negative consequences for the flow of traffic. In the other case involving positive acceleration, in contrast, Driver A is likely to dangerously overtake because she estimates the TTC of the approaching vehicle to be longer than it actually is. This TTC overestimation could reduce traffic safety.

We propose to exploit a time-honored and robust phenomenon of TTC estimation, namely, the size–arrival effect [23,24,25,26,27], to enhance the CMS mirror image in order to compensate for the inadequate consideration of acceleration of observers. The size–arrival effect refers to a perceptual phenomenon in which observers mistakenly factor the retinal size of objects into their TTC estimations: large objects are estimated to arrive earlier than small objects that approach in the same environment and at the same speed. In a conventional mirror, the retinal size of an approaching object increases as it approaches the observer. In CMS, this needs not be so; we can manipulate the projected size of the approaching object. Thus, if we increase the projected size of an approaching, accelerating object viewed on the monitor of a CMS by more than what would be the case in reality, we should be able to compensate for any error induced via acceleration by changing the displayed size accordingly. In other words, if an observer typically judges the TTC of a positively accelerating object to be longer than it actually is and we significantly increase the projected size of that object in a CMS during its approach, the observer should shorten the TTC estimate according to the size–arrival effect. Thus, it is conceivable that for a positively accelerating vehicle, its projected size can be inflated such that the observer no longer overestimates the TTC or does so substantially less. For real decelerating objects, the corresponding retinal size also increases as the object approaches the observer; however, this occurs at a significantly lower expansion rate. In a CMS, we could reduce the expansion rate to likewise improve judgment accuracy.

We conducted two experiments to determine if such a compensation for the TTC estimation error for accelerating objects is, in principle, possible by enhancing the mirror image of an approaching car viewed in a CMS. In the first experiment, we contrasted TTC estimations for positively accelerated and constant-speed car approaches with and without projection-size enhancements. We explored two types of size enhancements: a gradual increase in size and a sudden increase in size, the latter of which we expected to boost the effect of enhancing the TTC estimation by attracting more attention. Since the gradual increase in size was, however, more effective, we implemented only this type of size enhancement in the second experiment. The second experiment investigated whether the compensating effect of mirror-image enhancement also applies to negative acceleration.

## 2. General Materials and Methods

### 2.1. Stimuli and Task

**Figure 1:**
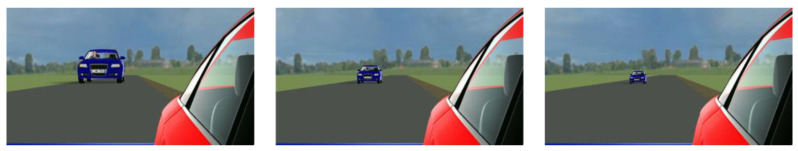
The three different vehicle’s sizes in Experiment 1 are depicted at a distance of 38.88 m from the reference line. On the left, the vehicle is enlarged to three times its original size, in the middle, it is enlarged to 1.5 times its original size, and on the right, the vehicle at its original size.

**Figure 2:**
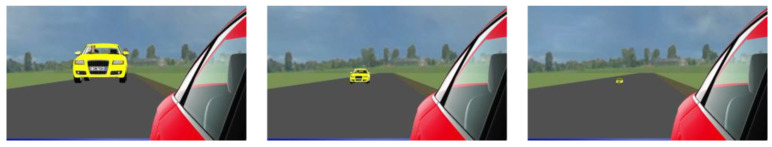
The three different vehicle’s sizes in Experiment 2 are depicted at a distance of 30 m from the reference line. On the left, the vehicle is enlarged to three times its original size, in the middle, the vehicle is at its original size, and on the right, the vehicle is reduced to one-third of its original size.

The stimuli shown on a computer screen depicted a visual road scene containing an oncoming passenger car as it would appear in the driver’s side rear-view mirror of the subject’s vehicle. The oncoming car travelled either at a constant speed or accelerated (positively in Experiment 1 and positively and negatively in Experiment 2). The scene consisted of a 10 m wide stretch of road with an asphalt texture which was flanked by green shrubberies, and the lighting simulated a clear day with a subtle blue sky. The subject’s vehicle was in a stationary position at the side of the road. A portion of the rear end of the subject’s vehicle was visible in the side monitor. A blue line was painted across the road 3.5 m behind the rear end of the subject’s vehicle such that it appeared at the very bottom of the screen, serving as a reference line for TTC estimation. The approaching vehicle moved along the road track 6.0 m from the side of the subject’s vehicle (the distance was measured from the midpoint of each car) and was 1.97 m wide, 1.50 m high, and 5.0 m long. Note that the size of the approaching car was intentionally altered in some experimental conditions in addition to the naturally occurring retinal change in the car’s size due to its approach. For constant-speed approaches, the car’s physical size was maintained throughout a trial and was presented in either its original, increased, or decreased size. For non-constant-speed approaches, the car’s size was kept either at its original size or was (a) increased suddenly or gradually (depending on experiment) for positive accelerations or (b) decreased for negative accelerations over a trial. In the cases in which the approaching vehicle had undergone an increase in size, it was expanded relative to a central point at the vehicle’s front, resulting in lateral, backward, and upward expansions of its projected image. In the case of a decrease in size, this also occurred from the central point outward. In Figure 1 and Figure 2, both experiments are depicted, along with representations of the projected sizes.

In each trial, the approaching vehicle was always visible for 2.0 s before it was occluded from the scene. After the occlusion, the empty road scene was still visible, and the subjects’ task was to estimate the time at which the front of the vehicle would have reached the blue reference line if it had continued its motion in the same fashion as observed before the occlusion (a prediction-motion task [28]). That is, they were asked to consider whether the vehicle had accelerated or moved at a constant speed. If the vehicle had accelerated before the occlusion, they should expect it to continue coasting or accelerating in the same manner. They were instructed to indicate their judgment by pressing the spacebar at the precise moment at which the blue reference line would have been reached. The scene did not include any audio. Each traffic scene for both experiments was created using Vizard 7, a 3D scenario development software.

### 2.2. Apparatus

The subjects conducted the experiment at home, using their own devices and the experimental software PsychoPy [29] (version numbers: 2022.1/2). In the experimental software, the participants adjusted the display settings to ensure that regardless of the device they were using, all subjects were presented with stimuli at a standardized size of 22.0 by 12.2 cm, mirroring the dimensions of a typical CMS monitor. The subjects were instructed to sit upright and to maintain a self-measured distance of approximately 60 cm to the screen during the experiment. Accordingly, the visual angle suspended by the display was 20.78° in width and 11.61° in height.

## 3. Experiment 1

To test the hypothesis that the size–arrival effect can be exploited to counteract deficiencies in the perception of acceleration, we varied the acceleration pattern (constant velocity and positive acceleration), vehicle’s size/size enhancement (depending on the acceleration pattern), initial velocity, and actual TTC.

### 3.1. Subjects

Nineteen psychology students (14 female, 5 male) with a mean age of 22.32 years (*SD* = 2.06 years) participated in the experiment for partial course credit. They were all recruited during a seminar at the Department of Psychology in Mainz. The study was conducted in line with the ethical standards of the Local Ethics Board of the Department of Psychology of Mainz University. Since voluntary participation on a fully informed basis and anonymity were assured, and there was no risk for physical stress or disadvantages due to group assignment, the ethics board deemed the approval of this experiment unnecessary. Out of the 19 subjects, 18 reported normal or corrected-to-normal near vision. The analysis included only the data from the 18 subjects with (normal) corrected near vision.

### 3.2. Design

We presented an approaching vehicle with two *acceleration levels*, 0 m/s^2^ (the constant-velocity approach) and +3.0 m/s^2^ (the accelerated approach). An overview of the experimental conditions regarding vehicle’s size and enhancement is depicted in Table 1. For the constant-velocity conditions, there were three *size variations*: (a) the vehicle remained at its original size (100% size), (b) the vehicle’s size was increased by a factor of 1.5 (150% size), (c) the vehicle’s size was increased by a factor of 3 (300% size). As described above, the size of the vehicle remained constant throughout the trial for the constant-velocity conditions. For the accelerated approaches, there were three *size enhancements*: (a) the vehicle remained at its original size without any size changes (original size), (b) the vehicle’s size suddenly increased to three times its original size at the midpoint of the visible time interval (sudden enhancement), (c) the vehicle’s size gradually increased from its original size to three times its size during the visible time interval (gradual enhancement). In summary, we presented three types of constant-speed and three types of accelerated car approaches, all of which were fully crossed with three *actual TTCs* (1.0, 2.0, 3.0 s) and three *velocities at occlusion* (*v_occ_* = 30, 50, 70 km/h). This resulted in 54 different driving profiles with occlusion distances ranging from 8.33 to 58.32 m. The initial starting distances were due to the different driving profiles within a range of 20.49 and 104.70 m.

The resulting 54 unique experimental conditions were presented six times, yielding a total of 324 experimental trials for each subject. They were preceded by 10 training trials to familiarize the subjects with the task.

### 3.3. Procedure

**Figure 3:**
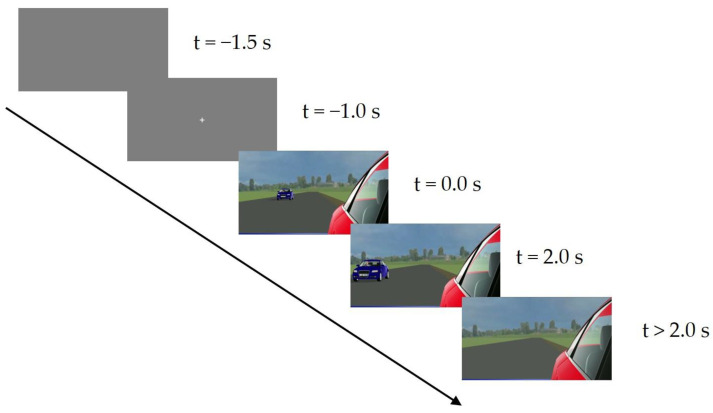
The subjects initiated a trial by pressing the spacebar. A gray background was presented for 500 ms, followed by a white fixation cross in the center of the screen for 1 s before the stimulus video appeared. After the vehicle had disappeared, the subjects indicated their TTC estimation by pressing the spacebar. The trial ended with the TTC estimation, and the subjects initiated new trials at their own pace.

At the beginning of the experiment, we instructed the subjects to imagine themselves in the perspective of a driver observing an approaching vehicle in a CMS monitor positioned close to and in lieu of a traditional side-mirror in their stationary car. The approaching vehicle would move at various constant speeds and would accelerate some of the time such that the respective same TTCs would result for all velocity conditions. The subjects first completed 10 randomly selected training trials to familiarize themselves with the road scene and the task. Subsequently, all experimental trials were conducted in different, random orders for each subject. The trial procedure is illustrated in Figure 3. After completing half of the experimental trials, the subjects were instructed to take a break lasting five minutes. Following the experimental trials, the subjects were asked to explain the strategies they used to estimate the TTC and whether they encountered any difficulties in making their estimates. Demographic information was also collected.

### 3.4. Results

In this experiment, we investigated situations with constant speed and positive acceleration, all with and without size variations/enhancements. We expected that a disproportional increase in the size of the projected vehicle’s size would lead to a shorter TTC estimation due to the size–arrival effect, both for constant-speed and accelerated approaches. For the accelerated approaches, we further expected this effect to be particularly pronounced at longer actual TTCs for which the deviation between first-order TTC estimates and the actual TTC is largest, as the estimation error for accelerating objects without size enhancements typically increases with an increasing actual TTC. For each combination of participant and experimental condition, we excluded extreme data points according to a Tukey criterion of three interquartile ranges below the first or above the third quartile, which affected 2.28% (*n* = 133 data points) of the *n_total_* = 5832 data points. Next, we aggregated the data per experimental condition.

**Figure 4:**
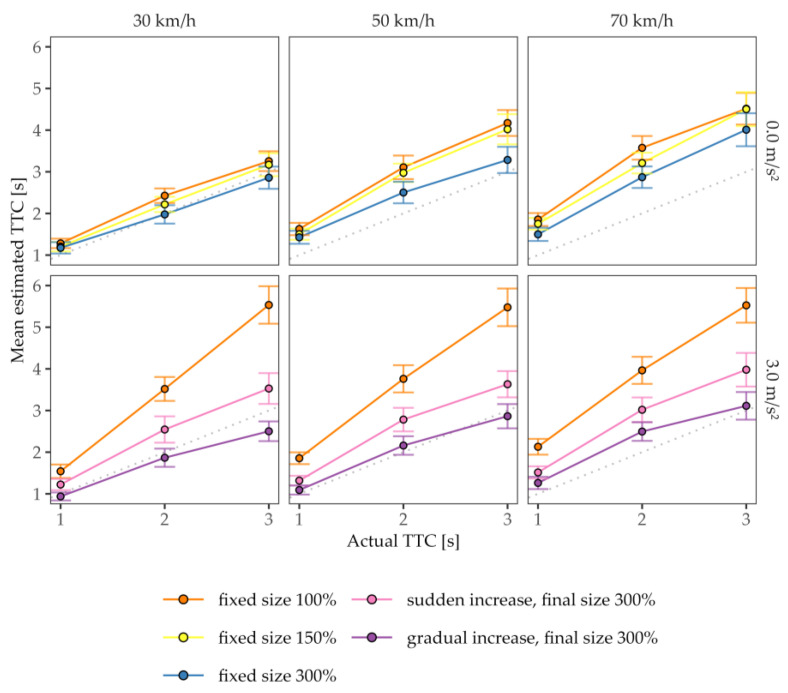
Mean TTC estimates as a function of the actual TTC values. Rows represent the acceleration level *a*, and columns indicate the velocity at occlusion *v_occ_*. The gray dashed line represents a perfect estimation of the contact time. Colors represent the variations of vehicle’s size/size enhancement. Error bars indicate ±1 SE of the mean.

To analyze the hypotheses, we conducted two separate repeated-measures ANOVAs (rmANOVAs) based on the mean TTC estimations for conditions with a constant velocity and for conditions with acceleration; each of these included the actual TTC, the velocity at occlusion, and the vehicle’s size/size enhancement as independent variables. All statistical analyses were interpreted with a significance level of α = 0.05 and were performed using RStudio 2023.06.1. Where applicable, we conducted pairwise paired-sample *t*-tests with Bonferroni corrections for follow-up comparisons. Figure 4 shows the mean estimated TTC as a function of the actual TTC.

For constant-velocity approaches (see Figure 4, upper row), as expected, the velocity and the change in the projected size affected the TTC estimations. The rmANOVA (see Table 2) confirmed that the TTC estimates decreased significantly as the projected size of the vehicle in the CMS increased (main effect of vehicle’s size), in line with the size–arrival effect. The mean estimated TTCs were the shortest for the 300% size condition (*M* = 2.40 s, *SD* = 0.96 s) and differed significantly from the 150% size condition (*M* = 2.73 s, *SD* = 0.91 s; *t*(17) = 4.38, *p_bonf_* < 0.001, *d_z_* = 1.03) and from the original-size condition (*M* = 2.87 s, *SD* = 0.90 s; *t*(17) = 4.88, *p_bonf_* < 0.001, *d_z_* = 1.15). The TTC estimates for the original-size cars did not significantly deviate from those for the medium-sized cars (*t*(17) = 2.50, *p_bonf_* = 0.069, *d_z_* = 0.59), suggesting that the 150% size condition did not provide enough magnification to elicit the size–arrival effect. The size–arrival effect was substantially more pronounced for longer actual TTCs than for shorter actual TTCs (size × actual TTC interaction) and for higher than for lower constant speeds (size × constant velocity interaction). In Experiment 1, the visual angle of the car (i.e., the vehicle dimensions shown on the screen) was not only influenced by the variation of the vehicle’s size/size enhancement but also by the distance of the car. The distance between the car and the observer at the start of a trial and at occlusion, for instance, covaried as the product of the constant speed and the actual TTC. As slower vehicles were, on average, occluded at a closer distance (corresponding to a larger visual angle) than faster ones, the significant main effect of constant speed as well as the interaction effect for constant speed and for the actual TTC may also be attributed to the size–arrival effect or to a distance bias [27,30], which predicts longer TTC estimates for objects at a greater distance than for close objects. This was the cost of varying the approach velocity. The three-way interaction did not reach significance.

For the accelerated approaches (see Figure 4, lower row), the TTC estimates for the condition with the original size descriptively followed a first-order pattern (overestimation of contact time increases with actual TTC). Across the enhancement conditions, the estimated TTCs increased significantly as a function of the actual TTC and the velocity at occlusion, which is again in line with a size–arrival effect or a distance bias.

The mean estimated TTCs for both the sudden (*M_error_* = 0.62 s, *SD_error_* = 1.05 s) and the gradual enhancement (*M_error_* = 0.03 s, *SD_error_* = 0.83 s) conditions showed, on average, smaller estimation errors (estimated-actual TTC) than for the condition without enhancement (*M_error_* = 1.70 s, *SD_error_* = 1.19 s). The effect of the size enhancement was confirmed via the rmANOVA on the mean estimated TTCs for the accelerated approaches (see Table 3). All three post hoc *t*-tests comparing the enhancement conditions reached significance (all *p_bonf_* ≤ 0.013), with the gradual enhancement reducing the TTC estimations more strongly than the sudden enhancement compared to the condition without enhancement (*t*(17) = 3.27, *p_bonf_* = 0.013, *d_z_* = 0.77). Hence, the size enhancement generally mitigated the first-order approximations significantly, and, in particular, the gradual enhancement proved to be effective. A look at the effect size of the size enhancement reveals that the manipulation had a rather strong effect. Taken together with the significant interaction between the enhancement and the actual TTC (see the diverging lines in Figure 4, bottom row), these effects are perfectly consistent with the interpretation that the subjects used the size cue to compensate for the errors induced by utilizing the first-order approximation for accelerating cars, as is common in ordinary viewing without size enhancement.

In summary, the results suggest that size manipulations have strong effects. These effects may be unintended during trials with constant velocity; in trials of positive acceleration, however, they can effectively reduce TTC estimation errors. Based on these encouraging findings, we conducted a follow-up experiment to explore the applicability of the gradual enhancement approach to situations involving negative acceleration.

## 4. Experiment 2

Our ability to judge the TTC of a decelerating object, despite its importance in many traffic situations, seems to be at least as poor as our ability to judge accelerating objects, e.g., [4]. Thus, artificially decreasing the size of decelerating vehicles should be as beneficial as increasing the size of positively accelerating vehicles. We combined both in Experiment 2. Also, to test whether the size manipulation can effectively alter the first-order strategy, we designed the stimuli such that the distance and velocity at occlusion were identical for all conditions.

### 4.1. Subjects

Forty-five students (42 female, 3 male) with a mean age of 23.04 years (*SD* = 3.64 years) participated in the experiment for partial course credit. They were all recruited via a seminar at the Department of Psychology in Mainz. They indicated that they had normal or corrected-to-normal near vision. Forty-one of the subjects had a valid driver’s license. Data from one subject were excluded as the subject’s responses indicated that the instructions had not been followed (TTC estimations of approximately 30 s in each trial). Therefore, the analysis included data from 44 subjects.

### 4.2. Modification of Stimuli

The motion pattern of the approaching vehicle was slightly modified due to feedback from some subjects in Experiment 1, who reported that they sometimes perceived the vehicle to change in size but to barely move. Thus, instead of applying acceleration throughout the entire 2.0 s approach interval as in Experiment 1, in Experiment 2, the vehicle moved at a constant speed for the initial 0.5 s before acceleration was applied for the remaining 1.5 s until occlusion. A second change was made to the color of the approaching vehicle. For better visibility, the vehicle color was changed from blue to bright yellow in Experiment 2.

### 4.3. Design

Based on the results from Experiment 1, only the threefold enlargement in the projected size (in the constant-velocity trials) and gradual enhancement (in the acceleration trials) were further investigated as they showed the most promising reduction in the TTC estimation error. Additionally, this experiment also examined negative acceleration. In order to achieve identical occlusion distances for all three types of acceleration (positive, negative, and none), it was necessary to adjust the acceleration magnitude and velocity at occlusion.

We presented the approaching vehicle with three *acceleration levels*, 0 m/s^2^ (constant-velocity approach), +2.0 m/s^2^ (accelerated approach), and −2.0 m/s^2^ (decelerated approach). An overview of the experimental conditions regarding vehicle’s size and enhancement is provided in Table 4. For constant velocities, there were three *size variations*: (a) the vehicle remained at its original size (100% size), (b) the vehicle’s size was decreased to a factor of one-third of its original size (33% size), and (c) the vehicle’s size was increased by a factor of three (300% size). The size of the vehicle remained constant throughout a trial for the constant-velocity conditions. For the accelerated approaches, there were two *size enhancements*: (a) the vehicle remained at its original size without any size changes (original size), (b) the vehicle’s size gradually increased from its original size to three times its size during the visible time interval (in the case of positive acceleration) or decreased from its original size to one-third of its size during the visible time interval (in the case of negative acceleration). In summary, we presented three types of constant-speed and two types of accelerated car approaches, all of which were fully crossed with three *occlusion distances* (*D_occ_* = 30, 45, and 60 m) and two *velocities at occlusion* (*v_occ_* = 60 and 80 km/h). This resulted in 18 different driving profiles with actual TTCs ranging from 1.28 to 5.26 s. Overall, there were 42 experimental conditions (18 conditions with constant velocity and 24 conditions with acceleration), each presented 10 times, resulting in a total of 420 experimental trials, in addition to 10 training trials.

### 4.4. Procedure

The procedure was similar to that of Experiment 1. However, unlike in Experiment 1, in which all conditions were presented in a random order, the experimental trials were blocked according to the acceleration enhancement (one block with enhancement and one block without enhancement). Twenty-one of the subjects began with the block including the size enhancement for the accelerated approaches, whereas the other twenty-three subjects started with the block including the accelerated approaches only without size enhancement. The trials involving constant speed were presented in both blocks, with all three sizes of vehicle included. The subjects were not explicitly informed about the blocking.

### 4.5. Results

Beyond the replication of the size-enhancement effects found in Experiment 1, Experiment 2 also considered negative acceleration. In the case of negative acceleration (deceleration), we expected an analogous effect. That is, a reduction in the projected size should lead to longer TTC estimates. Unlike Experiment 1, in which the actual TTC was varied as an experimental factor, in Experiment 2, we decided to vary the occlusion distance. This modification enabled us to compare the TTC estimations between the conditions at a given distance without having to account for additional distance-dependent variations in the optical angle and thereby the projected size. Per participant and experimental condition, we excluded extreme data points according to a Tukey criterion of three interquartile ranges below the first or above the third quartile, and we subsequently aggregated the data per combination of subject and experimental condition. This affected 1.09% (*n* = 201 data points) of the *n_total_* = 18,480 data points.

**Figure 5:**
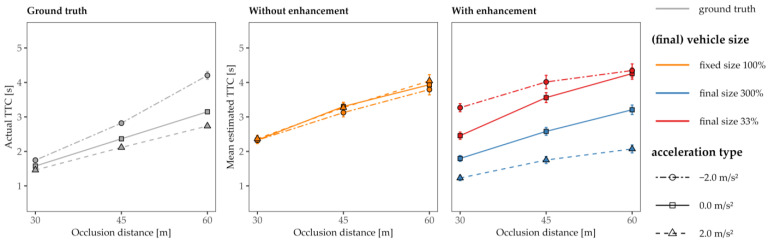
Actual and judged TTC values as a function of the occlusion distance for a comparison of the three acceleration conditions (decelerated *a* = −2.0 m/s^2^ circles; constant speed *a* = 0 m/s^2^ squares; and accelerated *a* = +2.0 m/s^2^ triangles). Left panel: the performance of an ideal observer (ground truth, gray color). Middle panel: approaches of cars at their original size (orange color). Right panel: approaches with size enhancement/variation. The vehicles in the constant velocity trials were increased (blue color, solid line) or reduced in size (red color, solid line). Accelerating vehicles were increased (blue color, dashed line) and decelerating vehicles were reduced in size (red color, dashed line). Note that the line types and shapes in all three panels indicate the acceleration rate. Error bars represent ± 1 SE of the mean.

Figure 5 shows the actual TTC (left panel), the mean estimated TTCs for all three acceleration levels (*a* = −2.0, 0, and +2.0 m/s^2^) for vehicles with an unaltered “original” size (middle panel), and the mean estimated TTCs for conditions with an altered size (right panel), each as a function of the occlusion distance. Since we implemented the same velocity and distance at occlusion for all three driving profiles, it was now possible to determine whether the TTC estimations followed a first-order pattern. Be reminded that a first-order estimation strategy is based on the vehicle’s distance and velocity at occlusion but neglects any acceleration. Note that a first-order TTC estimation strategy would only be appropriate for the constant-speed approach as there is *no acceleration information* that needs to be accounted for. If an observer uses such a strategy, the TTC estimations for constant-speed and accelerated approaches would be, in principle, identical. Although all three acceleration types produced different actual TTCs, it is striking that the TTC estimations showed only small deviations among the driving profiles, indicating that the acceleration for *a* ≠ 0 was not adequately taken into account without additional size enhancement, compatible with a first-order estimation. Figure 5 (right panel) further demonstrates that the TTC estimation were not solely reliant on the vehicle’s final size during occlusion. If this had been the case, the subjects would have judged the constant-speed and accelerated approaches with the same final size to be similar. Instead, they shortened their estimations for the decelerated approaches and lengthened their estimations for the accelerated approaches more strongly than for the constant-speed approach with the same final size, which highlights that gradual size variation also plays a major role in TTC estimation.

**Figure 6:**
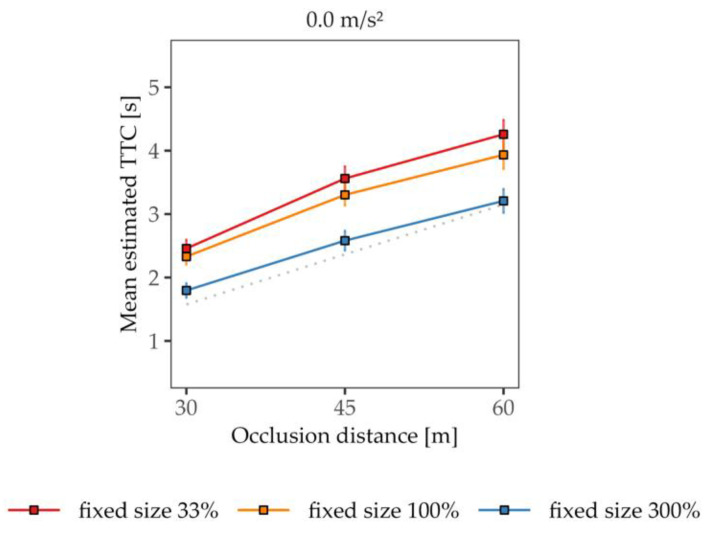
Mean estimated TTC as a function of the occlusion distance for constant-velocity trials. The gray dashed line represents the actual TTC (averaged across the different constant velocities). Error bars represent ± 1 SE of the mean.

Similar to the results from Experiment 1, the size–arrival-effect was observed for the constant-velocity trials (see Figure 6). As expected, a reduction in the vehicle’s size to a third of its original size led to significantly longer TTC estimations (*M* = 3.43 s, *SD* = 1.18 s) compared to the original-sized vehicle (*M* = 3.19 s, *SD* = 1.10 s), while an increase in the vehicle’s size by a factor of three led to significantly lower TTC estimations (*M* = 2.53 s, *SD* = 0.98 s) relative to the original-sized vehicle. The effect of the size adjustment was confirmed via a one-factorial rmANOVA for the constant-velocity trials (*F*(2, 86) = 84.28, *p* < 0.001, *η*^2^*_p_* = 0.66). All three post hoc tests comparing the size conditions reached significance (all *p_bonf_* < 0.001). Note that the final size in the 33% condition was only one-ninth of that of the 300% condition. The shift in mean TTC estimates quite nicely reflects the size proportion among the conditions, as is visible in Figure 6.

**Figure 7:**
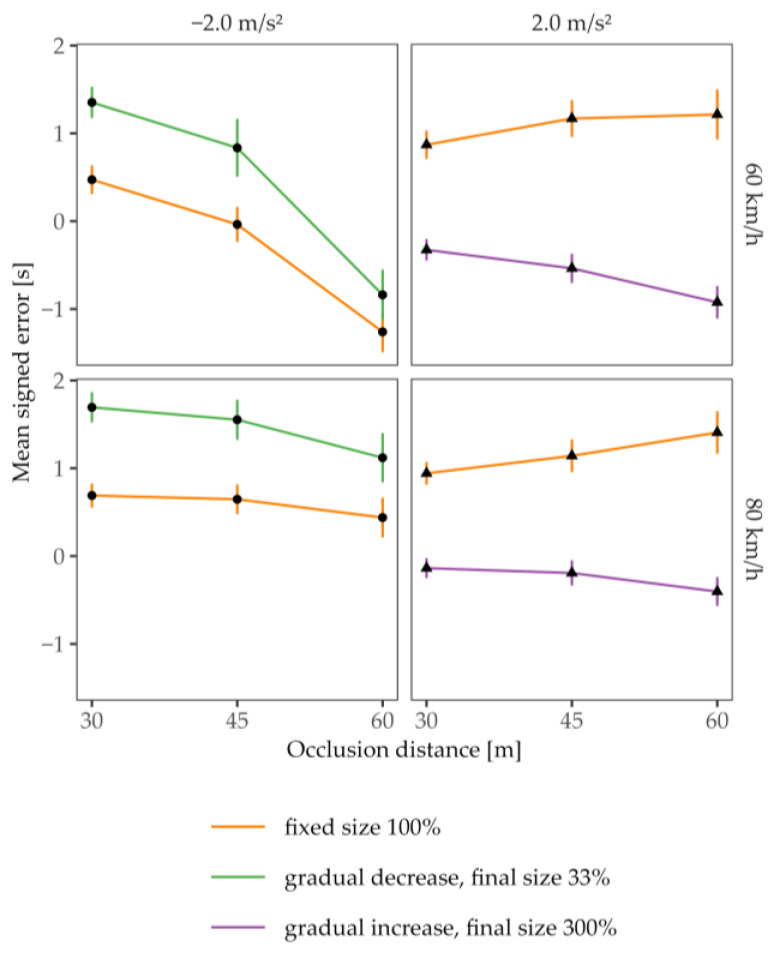
Mean TTC estimation error (estimated TTC minus actual TTC) as a function of the occlusion distance for accelerated approaches. Each row indicates an occlusion velocity. Shapes indicate the acceleration rate. Error bars represent ± 1 SE of the mean.

To examine whether the gradual size variation could compensate for errors due to first-order estimations for both positively and negatively accelerating vehicles, we contrasted the TTC estimates for conditions with and without enhancements for the different distances and velocities at occlusion. To this end, we analyzed the TTC estimation error, calculated as the signed difference between the mean estimated and actual TTC (mean signed error or constant error, e.g., [31]), for each of the two dynamic (*a* ≠ 0) driving profiles. Since the actual TTC covaried as a function of the acceleration rate, distance, and velocity at occlusion in Experiment 2, it was not possible to analyze the mean estimated TTCs. The mean signed error, in contrast, accounts for different actual TTCs and thus allowed us to compare the effect of the size enhancement among different distances and velocities at occlusion. As can be seen in Figure 7, the mean signed error decreased for the accelerated approaches with size enhancement (right column), compatible with a reduction in the overestimation of the TTC. In contrast, for decelerated approaches (left column), the gradual decrease in the vehicle’s size introduced larger mean signed errors. On average, the subjects lengthened their TTC estimates for decelerating cars when the vehicle “shrunk” during the approach, which is in line with the intended compensation for errors due to a first-order estimation. Since the size enhancements were qualitatively different for the two dynamic approaches, we conducted three-factorial rmANOVAs on the mean signed error for the accelerating and decelerating cars separately.

Table 5 presents the results of the repeated measures ANOVA (rmANOVA) for trials with positive acceleration, for which all effects were significant except the main effect of occlusion distance and the three-way interaction. The main effect for enhancement indicates that the presence of enhancement led to shorter mean signed errors. This effect is further amplified at lower speeds (the interaction effect of enhancement × occlusion velocity) and larger distances (an interaction between enhancement and occlusion distance). These findings are consistent with the size–arrival effect, suggesting that the subjects utilized the size manipulation/enhancement as a cue for estimating the TTC. In accordance with the ratio between distance and velocity at occlusion, which determined the actual TTC in Experiment 2, the subjects adjusted their estimations. However, their TTC estimates deviated more strongly from the veridical value at larger actual TTCs (interaction distance and velocity at occlusion).

Table 6 presents the results of the repeated measures ANOVA (rmANOVA) for trials with a decelerated approach, for which all main effects and the interaction effects for enhancement × occlusion distance and occlusion velocity × occlusion distance reached significance. The main effect for distance is consistent with the assumption that a change in velocity, i.e., deceleration, is less noticeable at a greater distance. This effect is particularly pronounced at lower speeds (the interaction effect of occlusion velocity × occlusion distance). The effect of enhancement was more profound for shorter than for larger distances (an interaction between enhancement and the distance at occlusion), which suggests that the gradual size reduction was more salient for the closer distance range.

To determine whether the enhancement had the same effect for dynamic (accelerated and decelerated) approaches as for constant-speed approaches, we aggregated the mean signed error across the different distances and velocities at occlusion and conducted an rmANOVA with the factors final size and acceleration. The latter factor included two levels (*a* = 0, *a* ≠ 0 with data for positive and negative accelerations). The significant interaction between final size and acceleration (see Table 7) confirms that the enhancement had a differential effect for dynamic and constant-speed approaches, suggesting that the final size was not the only cue being used in the modification of the TTC estimations for accelerated and decelerated approaches.

In summary, the results suggest that the size manipulations had the hypothesized effects and modified the TTC estimations. Increasing the size of accelerating vehicles and decreasing their size during negative acceleration changed judgments in agreement with the size–arrival effect.

## 5. Discussion

The objective of this study was to investigate the potential of camera–monitor systems (CMS) to compensate for perceptual errors in time-to-contact (TTC) estimations. When observers have to predict when a moving object will reach a given position, they make systematic errors of two kinds. On one hand, they fail to properly take acceleration information into account. They systematically overestimate the TTCs of positively accelerating objects and underestimate the TTCs of decelerating objects. In short, observers seem to neglect the acceleration after it is occluded and judge the TTC as if the object would continue to travel at a constant speed. We interpret this as a first-order estimation strategy. On the other hand, for a given actual TTC, estimates were affected by the retinal size of the approaching object. The TTC for small objects was relatively overestimated and the TTC for large objects was relatively underestimated compared to objects of intermediate size (size–arrival effect). Our goal was to find out whether the size–arrival effect can be exploited to compensate for the errors induced by acceleration.

If such compensation works, CMS would provide a unique opportunity to enhance the mirror image such that drivers can make more accurate TTC judgments as compared to regular rear-view mirrors. In the first experiment, we tested whether the two biases can indeed be played off against each other by inflating the projected sizes of approaching vehicles in a CMS when they accelerated suddenly or gradually during their approach. Indeed, image enhancements reduced the overestimation of the TTC values of positively accelerating vehicles. The compensation for the overestimation was more effective for the gradual size increase than for the sudden size increase. In the second experiment, we investigated whether a respective size diminution would likewise reduce the underestimation of the TTC observed for negatively accelerating approaches. In addition to increasing the size of accelerating vehicles, we reduced the size of decelerating vehicles projected in the CMS. As hypothesized, the data show that the respective gradual size changes reduced the estimation errors for both negatively and positively accelerating vehicle approaches.

### 5.1. The Potential Mechanisms behind the Size-Enhancement Effects

There are several reasons for which the size manipulations could have worked in the intended direction. The reasons differ according to whether or not one assumes the underlying processes to be, in principle, capable of accurate acceleration perception. If they are capable, the size manipulation could have provided access to this capability, for instance, by drawing the subjects’ attention to relevant second-order information associated with acceleration or deceleration, that is, the change in optical expansion relative to the object size over time. In principle, this information was readily available without image enhancement, but it might have been too subtle. Making the size alterations more extreme and thus more easily noticeable may have enabled the subjects to perceive and consider the acceleration in their TTC estimations. Such a mechanism has been proposed for auditory TTC information [14]. The sound of an internal combustion engine promoted the consideration of acceleration information in an audiovisual estimation of the TTC. In the present study, the size enhancement altered the second-order information consistent with a much stronger acceleration rate than what was actually present. However, we cannot distinguish whether the subjects merely recognized *that* the vehicle accelerated during the approach due to the size enhancement or whether they could also tell *how strong* the acceleration was. To investigate this further, it would be desirable to compare different acceleration rates with the same and different size enhancements. Even though we implemented two different (positive) acceleration rates with the same size enhancement in the two experiments, it is not possible to contrast the two conditions because fundamental changes between the experiments were made (regarding the final distance and thus the optical sizes of the objects), which limits their comparability.

If, in contrast, observers are taken to be unable, in principle, to accurately perceive the presented acceleration rate, which we think is more likely, then a different mechanism could explain the size enhancement effects. If the subjects only recognized *that* the vehicle accelerated, their adjustment of their TTC estimates could be explained by a heuristic strategy as follows: it is possible that the observers initially estimated the TTC as if the vehicle was traveling at constant speed. Subsequently, they revised their estimates by subtracting a fixed duration (e.g., 1 s) to account for the detected size change. In the case of size increases, such adjustment may reflect a safety strategy which has been observed in previous studies as a reaction to blurred vision or threatening sounds [18,19]. If this adjustment strategy was indeed used, the mean TTC estimates for the conditions with an increase in size would show a similar pattern as those without the enhancement but would be consistently shortened by a roughly constant value. However, our data do not provide evidence for such a pattern. It does not support the hypothesis of an unscaled adjustment of visual first-order TTC estimates in response to the detected acceleration. Moreover, the symmetric adjustments in the respective intended direction for both accelerating and decelerating targets rules out a mere safety strategy. Alternatively, it is possible that the adjustment of the first-order TTC estimations was guided by but not ultimately based on visual cues, where the subjects perceived a qualitative acceleration signal but adjusted their TTC estimates based on an a priori assumption regarding the level of acceleration. This assumption about the acceleration could have been derived from everyday experiences with dynamic traffic. This is in line with current theoretical perspectives on internal motion models, which are shaped via the experience of consistent factors, such as the effects of gravity [32,33,34].

In other words, prior assumptions about the expected retinal size for accelerated approaches might be accessed by the visual system. The size manipulations would capitalize on these priors. What could be the visual cue triggering the prior assumption about acceleration-related size? On one hand, the final visual angle subtended by the vehicle resulting from the distance and the vehicle’s size at occlusion might have been the relevant visual cue. This is, however, unlikely. We did control for this by manipulating the size not only in accelerated but also in constant-velocity approaches. If the size change had resulted in a mere additive modification of the TTC estimate, we would have obtained similar TTC estimates for the constant-speed and accelerated approaches with matching final optical angles. This was not the case, as underscored by the significant interaction between final size and acceleration (dynamic vs. constant-speed approach). On the other hand, the overall change in optical angle across a trial might have played a central role. In general, for decelerated approaches, the optical angle changed less over the course of a trial than for accelerated approaches because the distance travelled during the presentation was smaller. As a result, the change in the optical angle introduced by the gradual decrease in the vehicle’s size (“shrinking”) may have only been salient for observers when the decelerating car was comparatively close. This is in line with the TTC estimates for the decelerating “shrinking” car, which approximated those for the smaller cars (one-third-sized cars) approaching at a constant speed with increasing distance at occlusion. For the positively accelerating cars, the change in optical size across a trial was more pronounced in the enhancement condition due to (a) the longer travel distance but also due to (b) the larger final size of the vehicle. Hence, it is conceivable that the vehicle’s size “inflation” was more salient than its “shrinking” and was thus more stable across different distances at occlusion.

While it remains unclear how exactly the CMS image enhancement promoted the acceleration perception and/or the consideration of acceleration in the estimation of the TTC, it is worth emphasizing that this form of dynamically enhancing a vehicle’s size in the CMS appears to be particularly effective because the subjects responded to it strongly and intuitively. The subjects were not explicitly informed about the relationship between the size alteration and the driving behavior of the approaching vehicle. In contrast, static information about acceleration does not seem to improve TTC judgments; when acceleration was implied by the friction of a specific background surface on which an object was horizontally moving, observers failed to anticipate the object’s deceleration [16]. An explicit motion stimulus, e.g., a symbol or digital acceleration value provided by an external human-machine interface (eHMI), can inform an individual about acceleration and so that TTC estimates may be altered in the desired direction; however, unlike the intuitive size manipulation used in the current experiments, this requires cognitive processing and may be unintuitive. Ref. [35] instructed observers to interpret the illumination of a light band around the windshield of a car to indicate its acceleration, which did improve TTC estimation performance. However, in the design of CMS, size enhancement can capitalize on basic perceptual processes, which is desirable for smooth interactions with a CMS.

### 5.2. Caveats

When making repeated TTC judgments in the type of prediction-motion task we used, observers typically calibrate their responses as a function of the implicit or explicit feedback they may receive. Thus, the response baseline can shift according to past experience. As much sense as this type of perception–action attunement makes for most time-critical motor actions, it also poses a few problems when interpreting the results. For one, absolute errors are hard to interpret because the baseline can be shifted. Second, the exploitation of the size–arrival effect works when the approaching vehicle represents a standard size car, but it may not work, or may not work as well, when dealing with approaching vehicles that differ in physical size. Individual TTC errors may persist when a large truck is to be judged after a sequence of small sports cars. However, notwithstanding such effects, on average, the size enhancement should have the desired effect.

Within a prediction-motion paradigm, it is impossible to vary all relevant variables independently of one another. The final velocity before occlusion, final distance, extrapolation time, projected size at occlusion, and TTC are partially confounded. This problem cannot be completely solved. To work around the confounding problem, one can control different variables in separate experiments. Accordingly, in Experiment 1, we controlled for velocity at occlusion at the expense that the distance at occlusion covaried with the actual TTC and with the optical size at occlusion. In Experiment 2, we controlled for occlusion distance and final velocity at the expense that the retinal size covaried with the actual TTC. Given that in each experiment, the observers performed many trials, they may have caught on to the respective variable that was correlated with the TTC; accordingly, we cannot be entirely certain about the strategies observers may have used to accomplish the task.

A potentially more serious caveat concerns the question as to whether the results are generalizable to complex, everyday traffic situations. To obtain reliable TTC judgments, we decided to keep the subject’s vehicle stationary at a fixed position. During self-motion, the TTC estimates of other vehicles approaching on the passing lane will likely be more variable. Also, in all trials, only one vehicle approached upon which our observers could focus. Almost certainly, additional traffic will make TTC judgments less reliable. We know that other approaching vehicles, such as a truck in the lane next to the vehicle that is to be judged, produce a shift in the estimated TTC along the lines of a contrast effect (e.g., [36]). If we were to implement a size enhancement, should we then preserve such an effect and enhance all the approaching cars when multiple vehicles are present, or should only the most relevant vehicle be enhanced, and if so, shall it be the car nearest to the subject’s vehicle, or the one that accelerates most?

### 5.3. Implementation Challenges

The findings of this study have significant practical implications for improving driving safety through the use of CMS. We confirmed that the failure to adequately detect acceleration can be compensated for by exploiting the size–arrival effect. In principle, CMS open up opportunities to implement such compensations and to improve the accuracy of drivers’ TTC estimates, especially in interactions with accelerating or decelerating objects. However, a number of further requirements have to be met for their successful implementation. First, objective, sensor-based data about approaching vehicles must be measured and processed. This seems possible when the CMS can tap into the data streams collected for state-of-the-art driver-assistance systems. Second, the acceleration data of the identified vehicles must be translated into size changes in a closed-loop manner. That is, if an approaching vehicle first accelerates and then decelerates, the size of its image on the monitor would change accordingly. Depending on the acceleration behavior of its driver, this may look odd or even confusing. Alternating the deflation and inflation of a vehicle’s size looked acceptably natural for our stimuli with constant acceleration rates but may look rather strange when the acceleration and the related size changes are erratic. Many technical details need to be resolved as to how sudden the size manipulations should be. Third, a conflict resolution strategy needs to be implemented for those cases in which a size increase would occlude another vehicle, pedestrian, or object, depending on the traffic situation. Finally, it is not easy to determine the center of the vehicle’s expansion. During the pilot stages of the current experiments, we changed the center of expansion relative to which the vehicle grew in size. It looked odd when the wheels would dig into the pavement or disappear when expanding below its surface, and we accordingly chose an expansion point at the bottom of the car. This caused a slight size-induced drift of the car which was not critical in the current situation with only one approaching vehicle but might become problematic in more complex traffic situations. Manipulating a vehicle’s size within the camera image introduces various technical challenges that need further clarification. Nevertheless, as demonstrated, it seems to be a promising approach to improving TTC estimations.

## 6. Conclusions

Camera–monitor systems replace rearview mirrors with an analogous image on a monitor. This opens up the opportunity to process the image and enhance it with sensor-based information. Thus, CMS, together with the current advancements in automated traffic monitoring, provide a unique opportunity to enhance the “mirror image”, that is, the visual information that drivers can obtain about the rearward traffic situation. CMS open up a wealth of opportunities far beyond the limited enhancements that can successfully be applied by convex and aspherical mirrors. Our study provides proof of concept that perceptual errors, caused by a lack a sensitivity to acceleration information, can be successfully counteracted by altering the projected sizes of approaching vehicles in the monitor. As a next step, size enhancements should be tested in more complex situations using driving simulators to explore whether users also benefit from such size alterations in dynamic traffic situations.

## Figures and Tables

**Table 1:** Overview of the experimental conditions regarding vehicle’s size and enhancement in Experiment 1. Note that 100% refers to a naturally occurring change in retinal size.

Acceleration Rate	Vehicle’s Size/Size Enhancement
0 m/s^2^	100%, throughout each trial
	150%, throughout each trial
	300%, throughout each trial
+3.0 m/s^2^	100%, throughout each trial
	300%, sudden increase after 1.0 s of presentation
	300%, gradual increase during each trial

**Table 2:** Results of the rmANOVA on the mean TTC estimates for trials with constant velocity.

	*df* *_Num_*	*df* *_Den_*	$\tilde{\varepsilon }$	*F*	*p*	*η* ^2^ *_p_*
Actual TTC	2	34	0.55	117.56	<0.001	0.87
Constant velocity	2	34	0.69	56.98	<0.001	0.77
Size	2	34	0.80	19.31	<0.001	0.53
Actual TTC × constant velocity	4	68	0.99	13.02	<0.001	0.43
Actual TTC × size	4	68	0.74	5.53	0.002	0.25
Constant velocity × size	4	68	0.90	2.66	0.046	0.14
Actual TTC × constant velocity × size	8	136	0.77	1.22	0.303	0.07

Displayed are uncorrected numerator degrees of freedom (*df_Num_*), denominator degrees of freedom (*df_Den_*), the Huynh-Feldt multiplier for sphericity correction ($\tilde{\varepsilon }$), *F*-values, *p*-values and partial *η*^2^ (*η*^2^*_p_*).

**Table 3:** Results of the rmANOVA on the mean TTC estimates for trials with acceleration.

	*df* *_Num_*	*df* *_Den_*	$\tilde{\varepsilon }$	*_F_*	*p*	*η* ^2^ *_p_*
Actual TTC	2	34	0.53	115.54	<0.001	0.87
Occlusion velocity	2	34	0.70	27.22	<0.001	0.62
Enhancement	2	34	0.85	48.42	<0.001	0.74
Actual TTC × occlusion velocity	4	68	0.95	0.61	0.651	0.03
Actual TTC × enhancement	4	68	0.78	33.80	<0.001	0.67
Occlusion velocity × enhancement	4	68	1.00	0.88	0.481	0.05
Actual TTC × occlusion velocity × enhancement	8	136	0.56	2.19	0.071	0.11

Displayed are uncorrected numerator degrees of freedom (*df_Num_*), denominator degrees of freedom (*df_Den_*), the Huynh–Feldt multiplier for sphericity correction ($\tilde{\varepsilon }$), and the *F*-values, *p*-values, and partial *η*^2^ (*η*^2^*_p_*).

**Table 4:** Overview of the experimental conditions regarding the vehicle’s size and enhancement in Experiment 2. Note that 100% refers to a naturally occurring change in retinal size.

Acceleration Rate	Vehicle’s Initial Size	Vehicle’s Final Size/Size Enhancement
−2.0 m/s^2^	100%	33% (gradual decrease)
	100%	100%
0 m/s^2^	33%	33%
	100%	100%
	300%	300%
+2.0 m/s^2^	100%	100%
	100%	300% (gradual increase)

**Table 5:** Results of the rmANOVA on the mean signed error for trials with the accelerated approach.

	*df* *_Num_*	*df* *_Den_*	$\tilde{\varepsilon }$	*F*	*p*	*η* ^2^ *_p_*
Enhancement	1	43		106.92	<0.001	0.71
Occlusion velocity	1	43		35.87	<0.001	0.45
Enhancement × occlusion velocity	1	43		18.69	<0.001	0.30
Occlusion distance	2	86	0.56	0.45	0.530	0.01
Enhancement × occlusion distance	2	86	0.71	34.00	<0.001	0.44
Occlusion velocity × occlusion distance	2	86	1.02	7.17	0.001	0.14
Enhancement × occlusion velocity × occlusion distance	2	86	1.04	2.41	0.096	0.05

Displayed are uncorrected numerator degrees of freedom (*df_Num_*), denominator degrees of freedom (*df_Den_*), the Huynh–Feldt multiplier for sphericity correction ($\tilde{\varepsilon }$), and the *F*-values, *p*-values, and partial *η*^2^ (*η*^2^*_p_*).

**Table 6:** Results of the rmANOVA on the mean signed error for trials with the decelerated approach.

	*df* *_Num_*	*df* *_Den_*	$\tilde{\varepsilon }$	*F*	*p*	*η* ^2^ *_p_*
Enhancement	1	43		31.61	<0.001	0.42
Occlusion velocity	1	43		238.55	<0.001	0.85
Enhancement × occlusion velocity	1	43		1.42	0.240	0.03
Occlusion distance	2	86	0.86	72.33	<0.001	0.63
Enhancement × occlusion distance	2	86	0.76	5.07	0.015	0.11
Occlusion velocity × occlusion distance	2	86	0.84	99.94	<0.001	0.70
Enhancement × occlusion velocity × occlusion distance	2	86	0.78	0.49	0.567	0.01

Displayed are uncorrected numerator degrees of freedom (*df_Num_*), denominator degrees of freedom (*df_Den_*), the Huynh–Feldt multiplier for sphericity correction ($\tilde{\varepsilon }$), and the *F*-values, *p*-values, and partial *η*^2^ (*η*^2^*_p_*).

**Table 7:** Results of the rmANOVA on the mean signed error for trials with different final sizes and acceleration dynamics.

	*df* *_Num_*	*df* *_Den_*	*F*	*p*	*η* ^2^ *_p_*
Final size	1	43	103.74	<0.001	0.71
Acceleration	1	43	37.24	<0.001	0.46
Final size × acceleration	1	43	13.51	0.001	0.24

Displayed are numerator degrees of freedom (*df_Num_*), denominator degrees of freedom (*df_Den_*), *F*-values, *p*-values, and partial *η*^2^ (*η*^2^*_p_*).

## Data Availability

The data that support the findings of this study are available from the corresponding author upon request.
